# Editorial: Cross-talk of synaptic proteins in neurological diseases

**DOI:** 10.3389/fneur.2026.1880392

**Published:** 2026-06-08

**Authors:** Anil Annamneedi, Rushendhiran Kesavan, Gülcan Akgül, Rajkumar Vutukuri

**Affiliations:** 1 School of Arts and Sciences, Sai University, Chennai, India; 2 Children's Medical Center Research Institute, University of Texas Southwestern Medical Center, Dallas, TX, United States; 3 Izmir International Biomedicine and Genome Center, Dokuz Eylül University, Izmir, Türkiye; 4 Center for Neurogenetics, Feil Family Brain and Mind Research Institute, Weill Cornell Medicine, New York, NY, United States; 5 Institute of General Pharmacology and Toxicology, University Hospital, Goethe University Frankfurt, Frankfurt am Main, Germany

**Keywords:** Alzheimer disease, AMPA, autism spectral disorder (ASD), glutamatergic signaling, neurological disease, postsynaptic receptor regulation, presynaptic protein, S100B

The human brain relies on an incredibly complex network of synaptic connections, where precise communication between neurons is vital for everything from basic reflexes to high-level cognition. At the center of this network is the synaptic cleft-a tiny space where neurotransmitters, receptors, and scaffolding proteins interact in a finely tuned dance. The precise functioning of a synapse, occurs at a nanometre-scale spatial resolution and sub-millisecond temporal resolution, depends on a coordinated activity of presynaptic release machinery, postsynaptic receptor organization, cytoskeletal stability, glial modulation, extracellular matrix proteins, and activity-dependent plasticity. When this delicate balance is disrupted, it can lead to various neurological and neurodevelopmental disorders, such as epilepsy, Alzheimer's disease, and Autism Spectrum Disorder (ASD).

This theme collection compiles four landmark articles that bridge basic structural biology and human clinical pathology. Together, they showcase how molecular-level alterations at the synaptic junction scale up to network-level brain dysfunction.

**Figure 1:**
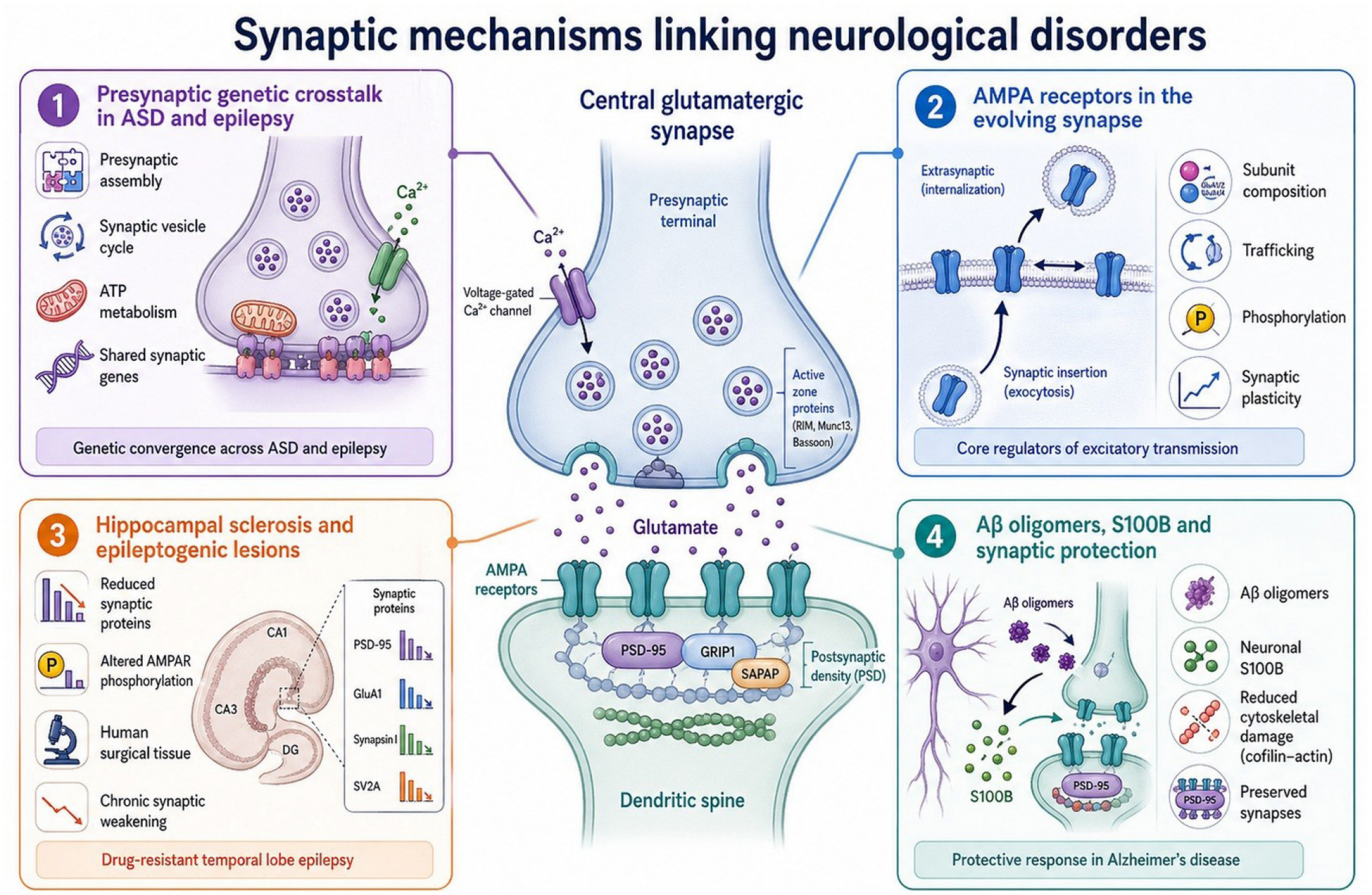
Synaptic mechanisms in different neurological disorders. The summary of the four articles of the special issue are included in this Research Topic, highlighting presynaptic genetic crosstalk in ASD and epilepsy, AMPA receptor regulation in excitatory synapses, altered synaptic protein expression and AMPAR phosphorylation in hippocampal sclerosis, and S100B-associated protection against amyloid-β-induced synaptic injury. The schematic was created by the authors with AI-assisted graphical support and manually reviewed for accuracy.

Each article in this Research Topic tackles a major obstacle in synaptic neurology, shifting paradigms from static descriptive pathology to dynamic molecular mechanisms (as detailed in Figure 1). One of the primary findings emerging from this Research Topic is the role of glutamatergic signaling, particularly AMPA receptors in maintaining synaptic functions. Francis et al. provide a comprehensive review on the significance of AMPA receptors, demonstrating how nanoscale movements, subunit compositions, and auxiliary subunit interactions (such as Transmembrane AMPA Receptor Regulatory Proteins, or TARPs) dictate synaptic strength. The rapid recruitment and disposal of AMPARs from the postsynaptic membrane enables synaptic strengthening and weakening, making them central mediators of long-term potentiation, depression, learning and memory. By mapping these basic structural dynamics onto psychiatric and neurological diseases, they clarify why disrupting AMPAR nanodomains leads to altered synaptic transmission across several disorders. Thus, this review on AMPA receptors explores both a mechanistic hub of synaptic plasticity as well as a vulnerable check point in neurological disorders.

This importance of synaptic proteins and the receptors is further supported by Oota-Ishigaki et al., who provide direct evidence from surgically obtained human brain tissue samples from patients with drug-resistant temporal lobe epilepsy associated with hippocampal sclerosis. A persistent bottleneck in epilepsy research is the reliance on rodent models, which do not fully replicate human pathology. Oota-Ishigaki et al. break through this barrier by analyzing actual surgically resected tissue from the epileptogenic zones of patients with drug-resistant focal epilepsy and hippocampal sclerosis. They show a chronic depletion of key synaptic proteins and a marked reduction in AMPA receptor (AMPAR) phosphorylation under chronic seizure conditions. This provides a direct molecular explanation for the clinically observed basal hypoactivity and hypometabolism in epileptogenic zones. Importantly, the study also connects febrile seizure history to enhanced AMPA receptor phosphorylation, suggesting that early life or clinical history may leave molecular traces on excitatory synaptic regulation.

While postsynaptic receptor regulation is widely concentrated in the field of synaptic neuroscience, Sharma et al. map out the shared genetic architecture of ASD and epilepsy, intentionally steering focus toward the often-overlooked presynaptic terminal. By performing *in-silico* analysis of literature based gene compilation and SynGO enrichment analysis, they demonstrate that shared genetic risk factors disrupt fundamental presynaptic operations, such as vesicular cycling, active zone assembly, and mitochondrial energy metabolism. This shifts the narrative of both disorders from postsynaptic channelopathies to a broader framework of presynaptic structural and metabolic dysfunction. By highlighting key disease associated genes, their loss and gain-of-function consequences of disease associated variants, the article supports a precision medicine approach wherein the therapeutic strategies may be developed aiming at synaptic dysfunction.

The fourth article, by Saavedra et al. extends the synaptic hypothesis into Alzheimer's disease and proteotoxic stress. The authors investigated the role of S100B, a calcium-binding protein mainly associated with astrocytes and neuroinflammation, but also expressed in neurons. In an Alzheimer's disease mouse model and primary neuronal cultures, they reported that oligomeric amyloid-β increases neuronal S100B expression, whereas monomeric or fibrillar amyloid-β does not have the same effect. Functionally, S100B partially reverses amyloid-β-induced cofilin-actin rod formation and rescues reductions in PSD-95 and active synapses in primary hippocampal neurons. These data support a model in which S100B acts as a protective chaperone-like response to amyloid-β oligomer toxicity, preserving cytoskeletal organization and synaptic integrity. This study is particularly valuable because it links proteostasis, neuron-glia signaling, cytoskeletal regulation, and synapse preservation within a single disease-relevant framework.

While addressing diverse clinical phenotypes, these articles are deeply interconnected by the themes of homeostatic plastic failures and tripartite synaptic communication. The presynaptic alterations identified by Sharma et al. (such as vesicle fusion machinery) directly determine the neurotransmitter release profiles that post-synaptically stimulate AMPARs. The theoretical mechanisms of AMPAR scaling and phosphorylation dynamics detailed by Francis et al. find their real-world clinical validation in the work of Oota-Ishigaki et al., where human temporal lobe epilepsy tissue displays a significant downregulation in the phosphorylation of these very same AMPAR subunits. Furthermore, the tripartite nature of the synapse is illuminated by Saavedra et al.'s exploration of S100B. While astrocytes typically regulate synaptic homeostasis, the upregulation of S100B inside neurons in response to amyloid β oligomers shows how cells adapt to preserve cytoskeletal integrity and prevent synaptic pruning. This directly aligns with the structural review by Francis et al., which highlights how amyloid β-induced cytoskeletal and synaptic loss leads to AMPAR internalization and cognitive decline.

As these papers propel the field forward, they point to several critical questions that must be addressed to translate these findings into clinical therapies. What are the exact spatial and temporal mechanics of presynaptic-postsynaptic crosstalk? How do the presynaptic genes identified in ASD/epilepsy (such as synapsins or calcium channels) dynamically coordinate with the postsynaptic AMPAR nanodomain adjustments reviewed here? Can we restore human synaptic protein loss *in vivo*? Having identified chronic synaptic protein depletion and hypometabolism in human epileptogenic zones, can we therapeutically reverse this process, or is it an irreversible structural scar of long-term seizure activity? How does patient history dictate synaptic biology? Since a patient's unique clinical history (such as early-life febrile seizures) can alter posttranslational AMPAR phosphorylation and lower the threshold for future hyperexcitation, how can we use these distinct molecular profiles to design personalized medicine strategies for epilepsy? How can we safely exploit S100B's chaperone function? S100B is a double-edged sword: at high extracellular concentrations, it acts as a pro-inflammatory RAGE agonist that worsens neurodegeneration, yet intracellularly, it acts as a neuroprotective holdase. Can we selectively boost intracellular/neuronal S100B chaperone activity in early-stage AD without triggering chronic neuroinflammation?

Ultimately, this Research Topic highlights that the synapse is not just a passive bridge, but a highly dynamic control center. By continuing to explore this molecular crosstalk, we move closer to developing targeted, synapse-specific therapies that can repair and restore the fragile networks of the diseased brain.

